# A qualitative analysis of self-management needs of adolescents and young adults living with perinatally acquired HIV in rural, southwestern Uganda

**DOI:** 10.1371/journal.pgph.0003037

**Published:** 2024-03-18

**Authors:** Scholastic Ashaba, Charles Baguma, Patricia Tushemereirwe, Denis Nansera, Samuel Maling, Alexander C. Tsai, Brian C. Zanoni

**Affiliations:** 1 Department of Psychiatry Mbarara University of Science and Technology, Mbarara, Uganda; 2 Global Health Collaborative, Mbarara University of Science and Technology, Mbarara, Uganda; 3 Department of Pediatrics, Mbarara University of Science and Technology, Mbarara, Uganda; 4 Center for Global Health and Mongan Institute, Massachusetts General Hospital, Boston, Massachusetts, United States of America; 5 Harvard Medical School, Boston, Massachusetts, United States of America; 6 Division of Infectious Disease, Department of Pediatrics, Emory University School of Medicine, Atlanta, Georgia, United States of America; 7 Department of Pediatric Infectious Diseases, Children’s Healthcare of Atlanta, Atlanta, Georgia, United States of America; 8 Hubert Department of Global Health, Emory University Rollins School of Public Health, Atlanta, Georgia, United States of America; National University of Singapore, SINGAPORE

## Abstract

The number of adolescents living with HIV remains high in sub-Saharan Africa with poorer HIV treatment outcomes among adolescents and young adults compared to individuals in other age groups. For adolescents and young adults living with perinatally acquired HIV (AYLPHIV), the transition from pediatric to adult HIV care is a particularly high-risk period. We conducted a qualitative study to understand self-management needs of AYLPHIV in rural, southwestern Uganda as they prepare to transition to adult HIV care in order to inform relevant interventions that can enable AYLPHIV acquire the necessary skills to manage their illness as they age into adulthood. We conducted 60 in-depth interviews with AYLPHIV (n = 30), caregivers (n = 20) and health care providers (n = 10) from the HIV clinic at Mbarara Regional Referral Hospital. We used an interview guide that focused on perceptions about transition to adult HIV care, challenges with transitioning, navigating HIV care, and self-management needs for AYLPHIV (from the perspectives of AYLPHIV, their caregivers, and health care providers). We used thematic analysis to identify themes related to AYLPHIV’s self-management skills. We identified several self-management needs that we grouped under two major themes; social support and empowerment for AYLPHIV to assume responsibility for their own health and to navigate adult HIV care independently. The sub-themes under social support were information support, instrumental support, and emotional support as the sub themes while sub-themes under empowerment included self-advocacy skills, interpersonal skills, self-care skills, and disclosure skills. Taken together, these findings indicate that AYLPHIV need to be supported and empowered to maximize their chances of successfully transitioning to adult HIV care. Support comes from peers and caregivers. AYLPHIV require knowledge about their HIV status and empowerment with different skills including: self-advocacy skills, interpersonal skills, self-care skills, and HIV status disclosure skills, in order to assume responsibilities related to independent HIV care.

## Introduction

HIV disproportionately affects adolescents and young adults in sub-Saharan Africa with about 88% of adolescents and young adults living with HIV residing in this region [1]. Furthermore, the number of adolescents and young adults infected with HIV in sub-Saharan Africa continues to rise due to the high risk of contracting HIV in this age group, with around 260,000 new infections reported among adolescents and young adults aged 15–24 years in sub-Saharan Africa in 2019 [2]. There are 1.4 million Ugandans living with HIV and, 5% of these are adolescents and young adults aged 15–19 years [3, 4]. Increased availability of antiretroviral therapy (ART) has enabled children born with HIV to survive into adolescence and young adulthood [5]. However, HIV treatment outcomes remain suboptimal in this age group, characterized by relatively high rates of virologic failure, disengagement from care, loss to follow up, and morbidity and mortality, compared to other age groups [6–8]. The risk of poor treatment outcomes is markedly elevated during the transition from pediatric to adult HIV care, especially for adolescents and young adults with perinatally acquired HIV (AYLPHIV) [9, 10].

One explanation that has been offered for the poor treatment outcomes during this critical transition period is that adolescents and young adults (who have, either since birth or since acquiring HIV, received HIV care through a much more hands-on, directive, and/or paternalistic model of pediatric care) often lack the necessary self-management skills to advocate for themselves in the adult HIV clinics and navigate care on their own [11–13]. Further challenges limiting the ability of the AYLPHIV to gain the necessary skills and become independent include HIV stigma, fear of HIV status disclosure, lack of self-efficacy, and poverty [10, 14–18]. In addition, AYLPHIV have to navigate the physiological and physical changes that occur during adolescence while trying to fit in with, and be accepted by, their peers [19, 20]. To optimize HIV treatment outcomes, AYLPHIV need to acquire social and psychological skills before they transition to adult care so that they can appropriately advocate for themselves and navigate adult health care settings [21–23].

In many countries in sub-Saharan Africa, health systems lack appropriate interventions or experience challenges implementing existing interventions to help AYLPHIV acquire the necessary skills to manage their illness as they age into adulthood [23]. AYLPHIV need to manage the symptoms of HIV, manage their treatment and side effects, make lifestyle modifications, and, in general, take responsibility for their own health [24]. Such self-management skills will help them successfully transition to adult care and live independently from their caregivers [25, 26]. Some of these skills include planning, goal-setting, problem-solving, resource activation, and gaining knowledge, all of which have been shown to be essential for self-management in chronic medical conditions [27–29]. Most interventions to promote self-management in chronic illness, however, were developed in high income countries and for other chronic medical conditions, and most HIV-specific interventions were developed for adult populations and may not be appropriate for AYLPHIV in sub-Saharan Africa [30, 31]. The few relevant HIV-specific interventions for adolescents living with HIV are not specific to AYLPHIV, nor are they focused on the transition period [32, 33].

Further, many HIV care facilities focus primarily on access to antiretroviral therapy, with little or no focus on attending to the psychological, social and behavioral aspects of the illness, such that many AYLPHIV age into adulthood without the skills necessary to manage the illness on their own [34, 35]. These skills are particularly vital for AYLPHIV in rural areas, where they often struggle with poverty, food insecurity, and limited educational opportunities in addition to managing a chronic stigmatized condition [17, 33, 36–38]. A precondition for developing relevant interventions to optimize AYLPHIV’s transition to adult HIV care, more research is needed about self-management of HIV in this population. In order to inform relevant interventions that enable AYLPHIV acquire the necessary skills to manage their illness as they age into adulthood, we conducted a qualitative study to understand the self-management needs of AYLPHIV in rural, southwestern Uganda as they prepare to transition to adult HIV care. In this qualitative study, we describe the self-management needs among AYLPHIV in a rural region of southwestern Uganda, elaborating the perspectives of AYLPHIV, their caregivers, and health care providers.

## Materials and methods

### Study setting

Participants were recruited from the HIV clinic attached to Mbarara Regional Referral Hospital (MRRH) in Mbarara city, Uganda. Mbarara city has a population of 195,013 (according to the 2014 census [39], the most recent data available) and is the primary business center of the region. Most people who access care from the MRRH HIV clinic live within the neighboring villages outside the city where they engage in subsistence agriculture, animal rearing and small scale trading to earn a living amidst challenges of water and food insecurity [40–42]. The prevalence of HIV in Mbarara district is estimated at 13%, more than twice the national prevalence of 5.8% [43]. The high prevalence of HIV in Mbarara district has been attributed to transactional sex practiced by sex workers in areas characterized by informal settlements, heavy alcohol consumption intersecting with sexual activity, and poverty with unequal access to financial resources within Mbarara city, which is the region’s commercial hub and a gateway to neighboring countries such as Rwanda and the Democratic Republic of the Congo [44]. The MRRH HIV clinic provides comprehensive HIV care including ART services, viral load monitoring and treatment of opportunistic infections. The adolescents and young adults’ HIV clinic is linked to the children’s clinic under the same pediatric HIV care team, which is distinct from the adults’ HIV care team. Although the Ugandan Ministry of Health provides guidelines for adolescents and young adults transitioning to adult HIV care [45], little preparation occurs at the study site due to understaffing. As a result, AYLPHIV who are transitioned to adult HIV care often lack the necessary self-management skills to manage their illness and navigate the adult HIV clinic on their own.

### Study participants and sampling

We conducted 60 in-depth interviews between November 2021 and April 2022 including 30 interviews with AYLPHIV, 20 interviews with caregivers (10 men and 10 women), and 10 interviews with healthcare providers. For AYLPHIV, we enrolled adolescents who were aged 15 to 24, fully aware of their HIV status, and receiving care at the MRRH’s HIV clinic. For caregivers, we enrolled both men and women who were caring for an adolescent living with perinatally acquired HIV aged 15–19 years or living with and supporting a young adult living with perinatally acquired HIV aged 20–24 years accessing care at MRRH. For health care providers, we enrolled clinicians, social workers, and counsellors who had at least 6 months’ experience caring for adolescents and young adults living with HIV at the MRRH HIV clinic. Every potential participant who was approached agreed to participate in the study.

Although previous research suggests that saturation can be reached after about 12 interviews [46], we conducted 60 interviews in order to ensure diversity of perspectives, purposively sampling both boys and girls (15–19 years) and young adult men and women (20–24 years), as well as male and female caregivers of diverse age groups. We were also interested in healthcare providers’ perspectives due to their expertise in the management of care for AYLPHIV. Our interest in participants who were living with HIV or who were knowledgeable about the challenges facing AYPLHIV and their transition to adult HIV care influenced us to use a purposive sample.

### Data collection, management and processing

Interviews were informed by a guide. We developed the guide based on the literature on self-management among AYLPHIV and based on input from professionals with expertise in the HIV care of AYLPHIV. The interview guide focused on perceptions about navigating adult HIV care, self-management skills, self-management needs, challenges of the transition to adult HIV care, and the ways in which health care providers and informal caregivers provided AYLPHIV with skills for transitioning to adult HIV care (S1 Text). The interview guide was developed in English and translated into the local language (Runyankore) and back translated to English to ensure concept reliability. The interviews were administered by three graduate level research assistants in either Runyankore or English according to the participants’ preference. On average, each of the interviews lasted one hour. Fifteen of the 60 participants interviewed opted to have the interview conducted in the local language. The audio recordings of the interviews were simultaneously transcribed and translated into English by the research assistants. Transcripts were reviewed by the research assistants and the first author (SA) to assess translation quality and fidelity.

### Data analysis

Following thematic analysis [47], two members of the research team (SA and PT) iteratively read the first eight transcripts, exchanged notes, discussed common concepts throughout the interviews, and collapsed related codes into an overarching category to create the codebook, which ensured the data’s rigor, credibility, and confirmability. As they read the transcripts, they noted emerging themes and sub-themes, sharing notes, and discussing emerging themes until consensus was reached. Through this process, the two team members were able to interpret and conceptualize internal meanings of the data through which themes (S2 Text) related to the self-management needs of AYLPHIV preparing to transition to adult HIV care were identified. The two coders then coded the first eight transcripts in duplicate, noting emerging themes and sharing coding strategies until consensus was reached. The remaining interviews were then divided between the two coders and coded independently. The data analysis process was guided by MAXQDA software version 20. For reporting, we followed the consolidated criteria for reporting qualitative studies (COREQ) guidelines [48].

### Ethical considerations

The study was approved by Mbarara University of Science and Technology Research Ethics Committee (MUST-2021-135), and clearance was obtained from the Uganda National Council for Science and Technology (UNCST) (#HS1697ES). Before enrolling in the study, all participants provided written informed consent. According to UNCST guidelines [49], two empowered adolescents (those under the age of 18 but who are empowered to assume responsibility for their own HIV care) were allowed to provide consent without the involvement of their caregivers. All participants received 25,000 Ugandan Shillings (approximately $7 at the time the study was conducted) for transport reimbursement. Audio recordings were immediately downloaded onto a password protected computer that was only accessed by two members (SA and PT) of the research team for the purposes of transcription. Participants’ names and other personal identifiers were removed from the transcripts before analysis to protect participants’ confidentiality.

## Results

Of the 30 AYLPHIV interviewed, 18 (60%) were male, the mean age was 20 years (standard deviation [SD], 2.2; range, 16–24), the mean duration on antiretroviral therapy (ART) was 15 years (SD, 3.8; range 6–21) (Table 1).

**Table 1:** Summary characteristics of the study participants.

Variables	Mean (SD) or number	%
**AYLPHIV (N = 30)**		
*Age*, *years*	20 (2.2)	
*Age started ART*	5.7 (4.2)	
*Years on ART*	15 (3.8)	
*Sex*		
Male	18	60
Female	12	40
*Religion*		
Christian	26	87
Moslem	4	13
*Marital status*		
Single	29	97
Married	1	3
*Education level*		
Primary	2	7
Secondary	15	50
Above secondary	12	43
*Transition status*		
Not yet transitioned to adult care	27	90
Transitioned to adult care	3	10
*Employment status*		
Formal employment	2	6
Informal employment	8	27
Student	20	67
**Caregivers (n = 20)**		
*Age*, *years*	47 (9.5)	
*Number of children*	3.9 (2.1)	
*Number of children in HIV care*	1.3 (0.7)	
*Marital status*		
Married	13	65
Single/separated/widowed	7	35
*Religion*		
Christian	19	95
Moslem	1	5
*Education level*		
Primary	15	75
Above primary	5	25
*Employment*		
Farmer	15	75
Business	5	25
**Healthcare providers (N = 10)**		
*Age*, *years*	39 (10)	
*Years providing HIV care*	7.2 (4.9)	
*Sex*		
Female	7	70
Male	3	30
*Marital status*		
Married	7	70
Single	3	30
*Employment designation*		
Clinician	5	50
Counselor	5	50

### Self-management needs of AYLPHIV

**Fig 1:**
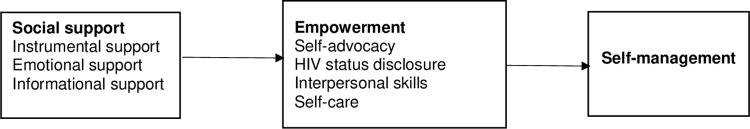
The social support and empowerment model.

In order to explain the self-management needs of AYLPHIV before their transition to adult HIV care, we used a logical model that illustrated how social support and empowerment link together and lead to self-management (Fig 1) whereby social support promotes empowerment. In support of this model, we identified themes relevant to the self-management needs of AYLPHIV, which we summarized under two major themes: empowerment skills and social support. Under the social support theme, the sub-themes included, instrumental support, emotional support, and informational support, (through disclosure of HIV status and through provision of information about their illness). Under the empowerment theme, the sub themes included self-advocacy skills, HIV status self-disclosure skills, interpersonal skills, and self-care skills.

### Social support

#### Instrumental support

Many AYLPHIV felt that parental support was a key factor that helped them (or would have helped them) assume the responsibilities of accessing care on their own. Participants noted that many AYLPHIV lack the support they need from an early age as they access care in the HIV clinic and are therefore unable to develop the skills to manage on their own as they age into young adulthood. Such scenarios were commonly reported among AYLPHIV who had lost their parents and who now lived with guardians; they mentioned that guardians did not pay much attention to the needs of AYLPHIV under their care. Key aspects of parental and caregiver support included: encouraging AYLPHIV to take their medicines and remain in care, providing information about the benefits of transitioning to adult HIV care, and giving them transportation fare to the clinic until they were financially stable.

***“**Although we say that for this person to transition they should be independent, still they need the support from their family members in terms of checking on them, sometimes providing them with transport [transport fare to the clinic] because even as adults sometimes they don’t have any money for transport. So they need to continue supporting them financially and psychologically to make sure that they remain in care until they are really stable [financially].”**—health care provider***

*“You know there are those [AYLPHIV] who lost their parents and live with their guardians, those people [guardians] be like ‘if you are going to the hospital, you go.’ They will not ask you anything, they don’t want to know whether you are sick or not, that doesn’t matter to them. You, the child should sort yourself out as long as they know that you are old, around 15 years.”—**young adult living with HIV, not yet transitioned to adult HIV care***

On the other hand, even some AYLPHIV who had grown up with their parents described how their parents were busy with work, too often assumed that AYLPHIV would find a way to survive, and did not offer much by way of support or encouragement in relation to HIV care. Some AYLPHIV reported that they just managed on their own, and that needing to fend for themselves compromised their ability to focus on their own HIV care.

*“They [parents/guardians] should protect us from any harm. You see some of our parents don’t care much about their children. They don’t value their children’s happiness. That is why they give them [children] away to other distant relatives to take care of them as if they are goats”.—**young adult living with HIV, not yet transitioned to adult HIV care***

***“**Parents should sit with their children and share about the good things at the adult clinic like the benefits they will get once they transition. Advise them to take their medicine well to have good health. They should not force them to change, they should instead persuade them to change and see the good things like becoming independent and making their own decisions, and becoming what they want to be.”—**young adult living with HIV, not yet transitioned to adult HIV care***

Participants agreed that AYLPHIV should have a steady income in order to be self-sufficient and manage their HIV care needs. Even though antiretroviral therapy was provided at no out of pocket cost in the HIV clinic, they still needed to pay for transportation to the HIV clinic (or any other healthcare facility for health care needs other than HIV care) and for non-medical physical needs, such as healthy, nutritious food as advised by their doctors. Some AYLPHIV expressed frustration with having to rely on their parents or other caregivers for financial support and felt they could not take responsibility for their own HIV care because they depended entirely on their caregivers. Participants mentioned that having a steady source of income is one way to help AYLPHIV gain control over their HIV care.

*“……to me independence has to be finances because in that we consider ability to feed yourself and of course you have to take a balanced diet, independence in a way that when you are needed at the clinic you are there, you are accessible in time, any time a doctor needs you, you are there. So, I see that independence has to do with finances.**”***

*—young adult living with HIV, failed transition to adult HIV care*

*“There is nothing that makes them [AYLPHIV] uncomfortable than asking their parents for each and everything. So at least if your child is not schooling try as much as you can to start something small that can sustain him/her. That will make him/her to become independent.”—**young adult living with HIV, not yet transitioned to adult HIV care***

#### Emotional support

Many AYLPHIV felt that transitioning to adult HIV care was associated with loneliness since they had few peers of their age in the adult HIV clinic that they could depend on for encouragement, which made it difficult for them to adjust to the new environment. AYLPHIV noted that seeking care with peers is very encouraging and motivating. In contrast, if they had transitioned alone, it would have been much more difficult; they felt that some could drop out of care due to lack of support from friends and peers. In the children’s clinic, AYLPHIV received support from their peers and could share their challenges and get advice, all of which helped them to stay retained in HIV care.

*“I think when they [AYLPHIV] are transitioning they [health care providers] should consider taking them [AYLPHIV] with their friends because if you take me alone I will be lonely and opt to go back to the children’s clinic to be with my friends because they [AYLPHIV] encourage us so much; so moving them [AYLPHIV] with their friends should be considered because support from friends matters a lot.”—**young adult living with HIV, not yet transitioned to adult HIV care***

*“They should be transitioned to the adult clinic as a group; it should not be for one child*. *At least when you are trying to transition*, *consider a group of adolescents*, *maybe like 5 of them at once*, *this will help them not be bored and they will not lose motivation in care***.”—caregiver**

#### Informational support

Some participants mentioned that AYLPHIV needed information about their illness, about antiretroviral therapy and the benefits of adherence, about how to navigate care when they transition to adult HIV clinics, and about how to take care of themselves and avoid engaging in health risk behaviors. They specifically felt that AYLPHIV needed to be previewed with information about the benefits of transitioning to adult HIV care so that they would be motivated to make the transition. If AYLPHIV were given this informational support, they reasoned, AYLPHIV would make the transition to adult HIV care and assume their new responsibilities with ease.

***“**They [AYLPHIV] should know everything about their illness, about their medicine, about their health, about the adult clinic. So the health care providers should share all the information they think will help these children get motivated. Give them time, listen to them and share everything freely with them. Surely that interaction will motivate them to transition without any challenge.”**—caregiver***

*“Well, they [AYLPHIV] need information about HIV illness, information about HIV medicine, how to deal with side effects and how to deal with challenges they might face as they change to the adult HIV clinic.”—**young adult living with HIV, not yet transitioned to adult HIV care***

A specific form of informational support mentioned by study participants was the need for AYLPHIV to be fully informed of their HIV status. Participants felt that AYLPHIV needed this information in order to manage their illness, live independently, and engage responsibly in care. They believed that recognizing HIV illness enabled them to understand the responsibilities that come with the illness and help them become committed to acquiring the skills necessary to navigate care on their own.

*“We [parents] should sit with our children and disclose to them everything and also give [them] the necessary counselling. When they go to other environments like secondary school while knowing their HIV status they will be able to take their medicine without fearing fellow students. Whatever circumstances they face, they will not stop taking their medicine.”—**caregiver***

*“They should tell the child [AYLPHIV] about their HIV*. *When I was young I would see my mother giving me medicine but not knowing why*, *she kept persuading me but at some point when I started coming [to the HIV clinic] by myself*, *one day the doctor told us that all of you here are HIV positive*. *Some of us did not take it easily and so they sent us to the counsellor for guidance*. *That helped me to adjust to my situation***”**

*—adolescent living with HIV not, yet transitioned to adult HIV care*

### Empowerment

#### Self-advocacy skills

Most AYLPHIV felt ill-equipped to manage HIV care on their own, including those who had not yet transitioned to adult HIV care. They described relying on their healthcare providers and caregivers to navigate care on their behalf beginning from an early age when they first began attending the children’s HIV clinic. They also stated that in the children’s HIV clinic, developing skills for navigating care independently was not a standard component of HIV care. Participants felt that AYLPHIV needed to be equipped with the necessary skills to build their self-esteem and assertiveness so that they could negotiate health care services for themselves in the adult HIV clinics.

*“They have to build skills of how to communicate, and how to do certain things so that they can negotiate a lot of things especially in their favor; they can learn to say ‘No’ or ‘Yes’ in their favor so that they are not harmed and [learn] other skills of survival, including seeking employment. We need to give them skills that will help them especially when it comes to communication, they should be as assertive as possible so that they are not trampled on.”—**health care provider***

*“I think the capability they [AYLPHIV] need here is the ability to speak up, that’s what I can say. This is because in the adult HIV clinic you will not find health care providers persuading you to talk [open up], so you have to be in a position to tell the doctors what is bothering you. They [AYLPHIV] need to have the potential to speak [up].—**young adult living with HIV, not yet transitioned to adult HIV care***

#### HIV status self-disclosure skills

Another critical skill mentioned by participants as being necessary for successfully transitioning to adult HIV care was that AYLPHIV needed to be able to disclose their HIV status to others in a way that was appropriate for their age, their relationship with the person to whom they disclosed, and the intended goals of the disclosure. Participants believed that disclosure of their HIV status to others reduced the stigma of HIV and, in general, enabled AYLPHIV to get support from peers, adhere to treatment, and more easily navigate care (because of the reduced fear of unintentional disclosure). They felt that disclosure also facilitated access to HIV care following the transition because AYLPHIV who have disclosed are more likely to share information with health care providers, enabling them to receive appropriate services.

*“They need to know that before they access HIV services, they should disclose to the doctors about each and everything about their health for them to access good health care services. They should be confident enough to disclose their health challenges to the health care providers and other trusted people for them to get proper services.”—**young adult living with HIV, not yet transitioned to adult HIV care***

*“But transitioning to adulthood it is not just the adult clinic but means meeting new people and disclosing [HIV status]; getting new jobs and explaining why you need day offs to go to the hospital. And most of the people [AYLPHIV] who embark on new journeys be it marriage, relationships, or a new job, when they fail to disclose, that is where non suppression starts from.”—**health care provider***

#### Interpersonal skills

Caregivers and providers stated that AYLPHIV required a set of character traits and interpersonal skills in order to capably manage their own HIV care in the adult clinic. For example, participants believed that AYLPHIV should learn perseverance and appreciate that they will encounter unexpected circumstances in life generally and in health care specifically. They felt that most AYLPHIV, particularly those in school who were trying to balance academics and HIV care, found it difficult to wait in line at the HIV clinic and often left without being seen. Participants also mentioned AYLPHIV needed appropriate communication skills so that they could approach and seek guidance from adults living with HIV and health care providers, which would make it easier to navigate care.

***“**For me I think they should be taught to be patient; like when they come to the hospital and find that the doctor has not yet arrived, let them learn to be patient until the doctor comes and works on them. So prepare them to be tolerant and patient, everything might not go the way they want. Teach them all about the adult HIV clinic procedures and since they [procedures] might be different from those at children’s clinic, let them learn to be patient in every situation**.”—caregiver***

*“Communication skills; they need to learn to communicate with people; they need to learn to communicate with their health care providers. Previously it is mum and dad who have been communicating on their behalf but this time around it is them [AYLPHIV] explaining themselves. In communication skills they should be able to express themselves, they need to learn to greet, learn to say sorry, learn to explain things in their own way, they need to ask where they don’t understand, they need to appreciate where it is due.**”—health care provider***

#### Self-care skills

Another self-management skill frequently mentioned was that AYLPHIV needed to know how to practice self-care. Participants felt that AYLPHIV should be educated about self-care, that they needed to love and appreciate themselves, which they believed was necessary for AYLPHIV to take care of themselves, both physically and mentally. Other aspects of self-care mentioned included routine tasks such as medicine storage, personal hygiene, and nutrition. Most participants suggested that AYLPHIV should be taught basic self-care skills prior to transitioning to adult HIV care, so that they could navigate HIV care on their own.

*“They have to take care of themselves better than the way the doctors are doing it to them. They should love themselves and take good care of themselves by taking well their medicine, eating well, keep happy, and think about their future so that whatever change it is, is for the good of their lives. I think that is what they should know”.—**young adult living with HIV, not yet transitioned to adult HIV care***

*“Teach them also to be careful with their health; they should not be involved in things that can put their health in danger because they look at themselves as adults so when they want to engage in sex they should use protection to avoid contracting STIs**.”—caregiver***

## Discussion

In this qualitative study of AYLPHIV, caregivers, and health providers in a rural region of southwestern Uganda, we identified self-management needs for AYLPHIV facing the transition to adult HIV care. We summarized these needs into two major themes: social support and empowerment. Social support included instrumental support, emotional support, and informational support. Empowerment included various skills around self-advocacy, HIV status disclosure, interpersonal interactions, and self-care. These themes are consistent with the self-management framework, which posits that self-management comprises numerous components that interact to enable positive HIV-related behaviors and outcomes [50, 51]. This also echoes the Social-ecological Model of Adolescent and Young Adult Readiness to Transition, which incorporates modifiable targets to improve transition including knowledge, self-efficacy, social support, and peer support [13, 52].

The role of social support in self-management is in line with earlier studies that have demonstrated the importance of social support in building resilience and self-management among AYLPHIV [53]. The three types of social support (instrumental, emotional, and informational) line up with the typology elaborated by Cohen and Wills [54]. AYLPHIV who have supportive networks of family and friends are more likely to have acquired necessary self-management behaviors, are less likely to miss clinic appointments, and often have a positive attitude towards life despite their illness [53, 55, 56]. Furthermore, emotional support enables AYLPHIV to overcome challenges of depression, anxiety, and stigma, which can interfere with their ability to engage in self-management behaviors [15, 18, 55]. Peer support groups have been noted to be helpful in promoting self-management behaviors such as increased self-confidence, adherence to ART, and overcoming fears associated with HIV status disclosure and stigma [57, 58]. Moreover, peer support groups provide a secure space for sharing experiences, exchanging information, and providing emotional support [59–62]. Instrumental support was seen as helping AYLPHIV transition to adulthood more generally, and lack of financial stability was viewed as a hindrance to the assumption of adult roles (including HIV care) [33, 63–65]. Having a stable source of income has been associated with transition readiness among AYLPHIV [66] while economic empowerment has been associated with improved HIV treatment outcomes among AYLPHIV [17, 67]. Finally, the informational support needed entails comprehensive knowledge of HIV, HIV transmission, and the role of ART adherence [68, 69], as well as sexual and reproductive health [70, 71].

Empowerment was viewed by participants as a necessary precondition for successfully transitioning to HIV care. Prior work has linked better outcomes to self-advocacy [51, 69, 71–75], HIV status disclosure [11, 66, 76–80], interpersonal skills [81–83], and self-care [50, 84–86]. AYPLHIV should be empowered with skills to enable them participate effectively in their care including making decisions related to their care, collaborating effectively with health care providers as they grow towards independence and assuming responsibility for their own HIV care [68]. It should be noted that empowerment and social support can also interact bidirectionally. For example, disclosure of HIV status can also open up opportunities for social support, which facilitates continued engagement in care [79, 87].

Our study findings should be interpreted bearing in mind the following limitations. First, the data were collected from a single HIV clinic in southwestern Uganda, and the results may not be generalizable to all AYLPHIV transitioning to adult HIV care in Uganda. However, we obtained different perspectives from a diverse group of AYLPHIV, caregivers, and health care providers. Second, most of the AYLPHIV participating in our study had not transitioned to adult HIV care. Their views may not represent true needs of AYLPHIV who have already transitioned to adult HIV care (whether successfully or unsuccessfully). Third, because we only conducted in-depth interviews, the participants’ perspectives may have been influenced by factors such as social desirability bias. Fourth, fifteen of our interviews were conducted in the local language (Runyankore) and then translated into English. As a result, the narrative of these interviews may not be accurate because some words in Runyankore have no exact translations into English. However, the research assistants and the first author (SA) are both fluent in Runyankore and English, which could have mitigated problems that could have affected the content in the Runyankore interviews.

## Conclusions

In order to assume responsibilities for their own health, AYLPHIV in sub-Saharan Africa need to be empowered with various skills including self-advocacy, self-care and interpersonal skills. The study results further show that that AYLPHIV need instrumental, emotional and informational support to better manage themselves and transition to adult care. These self-management needs highlight the potential significance of behavioral interventions with numerous components to help AYLPHIV transition to adult HIV care and assume responsibility for their health and HIV care [78, 88]. Potential interventions should target empowerment of the AYLPHIV in HIV status disclosure skills and as well as empowerment against HIV stigma especially internalized HIV stigma. Previous research has documented the role of a peer-led HIV self-stigma intervention in improving self-worth and wellbeing among young people living with HIV [89]. Other interventions should aim to financially empower AYLHIV through income-generating activities and vocational training to enable them to acquire skills to sustain themselves financially.

## Supporting information

S1 Text Interview guide. (DOCX)

S2 Text Themes and quotes related to the manuscript. (DOCX)
