# Supplementary material for: A qualitative analysis of self-management needs of adolescents and young adults living with perinatally acquired HIV in rural, southwestern Uganda
Source: PLOS Glob Public Health. 2024 Mar 18;4(3):e0003037. doi: 10.1371/journal.pgph.0003037 (PMC10947701; doi:10.1371/journal.pgph.0003037)
Supplement: S1 Text — (DOCX) [file pgph.0003037.s001.docx]

**Interview guide (Adolescents living with HIV)**

|  | **Perceptions, concerns, experiences and challenges related to transition from the children’s to the adults’ HIV clinic** | *Probes for RAs* |
| --- | --- | --- |
| 1 | What are some of the things that should be considered before AYLHIV change from doctors for children to doctors for adults in the HIV clinic | *Probe about age, knowledge about HIV, self-management, knowledge about illness, knowledge about medicines, expectations from the doctors* |
| 2 | What do you think can be done to ease the process of ALHIV changing doctors and start seeing doctors for adults living with HIV | *Probes: Probe on what they think the adolescents can do make the change process easier, what can the parents or care givers do to make the change process easier, what could the health care providers do to make the change easier.* |
| 3 | What do you think is difficult about changing from the children HIV clinic to begin accessing care in the adult HIV clinic? | *Probe*  *On each difficulty mentioned*  *Their ability to remain calm despite the difficulties*  *Ability to stand up for their own rights when things are not easy* |
| 4 | What challenges do adolescents and youth living with HIV face while changing from doctors for children to doctors for adults in the HIV clinic? | *Probes:*  *Probe on each challenge raised and why they think that challenge can hinder their transition process* |
| 5 | What challenges do AYLHIV face while interacting with health care providers doctors in the adult HIV clinic when they change care from the children’s to the adult HIV clinic | *Probe on each of the challenges raised*  *For those who have transitioned probe for specific challenges according to their personal experiences* |
| 6 | What challenges do AYLHIV face while interacting with adults patients living with HIV when they change care from the children’s to the adult HIV clinic | *Probe on each of the challenges raised*  *For those who have transitioned probe for specific challenges according to their personal experiences* |
| 7 | How can ALYHIV be helped to gain confidence in themselves in who they are as people living with HIV before ALYHIV before they change from doctors for children to doctors for adults? | *Probe on*  *Self-beliefs*  *Empowerment*  *Determination*  *Information*  *Encouragement* |
| 8 | How do you usually come up with solutions when you face problems associated with your health and HIV care? | *Probe*  *Specific problems mentioned and possible solutions*  *What can you do as an individual* |
| 9 | What should adolescents living with HIV know about their illness before they change from doctors for children to doctors for adults as they get older? | *Probes:*  *Probe on the ability to take of their illness, why it is important that they learn to take care of their illness before they change, what they should know about their medicines before the change, the importance of understanding their medicines before they change* |
| 10 | What should adolescents living with HIV know about accessing HIV care before they change from doctors for children to doctors for adults as they grow older? | *Probes:*  *Probe on the importance of understanding how to access care before they change, the kind of support they need before they change.* |
| 11 | What are some of the things adolescents living with HIV need to know about self-care before they change from doctors for children to doctors for adults in the HIV clinic? | *Probes:*  *Probe on physical care, emotional wellbeing, social care* |
| 12 | How should family members empower AYLHIV so that they can successfully change care from the children’s to the adult HIV clinic | *Probes:*  *Probe on the role of family in empowering the adolescents for this change of doctors, how can the family members work with health care providers and the adolescents to make the change easy* |
| 13 | How can health care providers empower AYLHIV to successfully change HIV care from the children’s to the adult HIV clinic older | *Probes: How should health care providers empower adolescents to smoothly change from child HIV clinics to adult HIV clinics*  *How should health care providers work with family members to ensure this change is smooth*. |
| 14 | What kind of preparation and training should ALYLHIV receive before they change from doctors for children to doctors for adults | *Probe on : What these trainings should comprise of, about what, at what age should they start, who should participate, who should facilitate these trainings, what would be the role of doctors and parents/guardians, how often should they happen* |
| 15 | What are some of the things that can be done to motivate AYLHIV to change from doctors for children to doctors for adults successfully | *For each factor mentioned probe why and who should be responsible to ensure that it happens* |
| 16 | What kind of information do AYLHIV need to equip them for a successful change from doctors for children to doctors for adults in the HIV clinic | *Probe*  *Information about HIV as a chronic illness*  *Information about HIV medicines*  *Information about adult HIV clinic*  *Information about continuity of care in the HIV clinic* |
| 17 | What is the importance of taking HIV medications for adolescents living with HIV? | *Probes:*  *Probe on where they learnt the information from, how those who don’t know can access this information* |
| 18 | What do adolescents and youth living with HIV need to know about their medicines before they change from the HIV clinic for children to the HIV clinic for adults? | *Probes:*  *Probe on what they need to know about their medicines, where they learn the information from, how those who don’t know can be helped to access this information* |
| 19 | How does making clinic appointments on their own help adolescents and youth living with HIV | *Probes:*  *The importance of making clinic appointments on their own, what they need to know about making clinic appointments, why they should know how to make clinic appointments on their own, where they learned information from, how those who don’t know can be helped* |
| 20 | What do adolescents and youth living with HIV need to know about their illness before they change from doctors for children to doctors for adults for their HIV care? | *Probes:*  *Where they learnt from what they know about their illness, how those who don’t can be helped* |
| 21 | How should AYLHIV be empowered to handle the health challenges related to their illness before they change from the children’s clinic to start their care in the adult HIV clinic | *Probe on how they learned to handle health challenges related to their illness, how adolescents going through similar challenges can be helped to handle their own challenges* |
| 22 | What capabilities do AYLHIV need to cope with HIV as they change from the children’s HIV clinic to the adults HIV clinic when get older | Probes  *Probe on how they can seek support from health care providers and peers, how to express their feelings freely, how to interact with peers and health care providers* , communication skills |
| 23 | What capabilities do AYLHIV need to cope with the change of health care providers doctors for children to the health care providersdoctors for adults when they get older | Probes  *Probe on how they can seek support from health care providers and peers, how to express their feelings freely* |
| 24 | How do you think you can handle problems that you may face during the process of changing from the children to the adult HIV clinic to continue care as an adult? | *Probes*  *Communication*  *Knowledge about HIV care process*  *Information about navigating HIV care*  *Social support systems*  *Self-disclosure* |
| 25 | What are some of the things that can make you believe in yourself that when you change from the children’s HIV clinic to the adult HIV clinic you will have no interruptions in your HIV care? | *Probes*  *Personal goals*  *Determination*  *Acceptance of status*  *Self-beliefs*  *Knowledge about HIV care* |
| 26 | How are you able to ensure that the challenges that you may face when you change from the HIV clinic for children to the HIV clinic for adults will not affect your ability to continue with HIV care? | *Probe on*  *Ability to focus on solutions*  *Determination to solve challenges*  *Confidence in self-confidence* |

**Interview guide (Health care providers)**

|  | **Perceptions, concerns and experiences of change of care from pediatric clinic to the adult clinic** |  |
| --- | --- | --- |
| 1 | What are some of the things that should be considered by health care providers before AYLHIV change from the pediatric HIV clinic to the adult HIV clinic? | *Probe about age, knowledge about illness, knowledge about the illness self-care skills, employment status, financial sustainability ,* |
| 2 | What do you think can be done to ease the process of change from health care providers for children to health care providers for adults in the HIV clinic for adolescents living with HIV? | Probes:  *How should be transition be handled*  *How should AYLHIV be empowered*  *What skills should be focused on to empower AYLHIV for transition* |
| 3 | As a health care provider what are your concerns about the change for adolescents and youth living with HIV from health care providers for children to health care providers for adults in the HIV clinic? | *Probes*  *Probe on each concern raised and their suggestions on what can be done to address these concerns* |
| 4 | Which skills do AYLHIV need to get motivated for a successful transition from pediatric to adult HIV clinic for continuity of care? | *Probes*  *Probe on any skills given on how it can motivate the adolescents and youth* |
| 5 | What challenges are AYLHIV likely to face as they change from pediatric to adult HIV clinic? | *Probe on challenges at individual level, challenges on family and peer relationships, challenges associated with facilities where they access care, challenges related to health care providers* |
| 6 | What should adolescents AYLHIV know about their illness before they change from health care providers for children to health care providers for adults? | *Probes:*  *HIV as a chronic disease*  *HIV medicines, side effects, benefits, adherence, retention in care* |
| 7 | What should AYLHIV know about accessing HIV care before they change from health care providers for children to health care providers for adults? | *Probes:*  *Probe on the importance of understanding how to access care before they change, the kind of support they need before they change.* |
| 8 | What skills do adolescents and youth living with HIV need to cope with the challenges of transition from the pediatric to the adult HIV clinic? | *Probe on how they can seek support from health care providers and peers, how to express their feelings freely* |
| 9 | As a health care provider working with people living with HIV how do you think you can empower AYLHIV to transition successfully and continue with care in the adult HIV clinic? | *Probes:*  *What skills can AYLHIV gain from the health care providers and the HIV clinic in general before transition, self-confidence* |
| 10 | How should family members empower AYLHIV so that they can successfully change care from the children’s to the adult’s HIV clinic | *Probes:*  *Skills that AYLHIV can gain from home and the community to ensure continuity of care after transition* |
| 11 | What challenges do you think AYLHIV may face while interacting with adults when they change care from the children’s to the adult HIV clinic | |
| 12 | What kind of preparation and training should AYLHIV receive before they change from health care providers for children to health care providers for adults? | *Probe on the types of preparation and training mentioned* |
| 13 | What are some of the things that can be done motivate AYLHIV to change from health care providers for children to health care providers for adults successfully | *Probes: Sensitization meetings, peer support, family support, health care provider support, health education* |
| 14 | How do you think AYLHIV can handle challenges that they may face during the process of changing from the pediatric to the adult HIV clinic? | Probes: Communicate with health care providers, support from health care providers, support from caregivers, peer support, health education |
| 15 | What skills do AYLHIV need to ensure that the challenges they face when they change from the HIV clinic for children to the HIV clinic for adults will not affect their ability to continue with HIV care? | *Probes*  *Communication*  *Knowledge about HIV care process*  *Information about navigating HV care*  *Social support systems*  *Self-disclosure* |
| 16 | What are some of the things you can do as a health care provider to make AYLHIV believe in themselves as they change from the children’s HIV clinic to the adult HIV clinic to avoid interruptions in their HIV care? | *Support, training, provide reading materials, health education, counselling* |

**Interview guide (Parent/guardians)**

|  | **Perceptions, concerns and experiences of change of care from pediatric clinic to the adult clinic** | *Probes for RAs* |
| --- | --- | --- |
| 1 | What are some of the things that should be considered before AYLHIV change from health care providers for children to health care providers for adults in the HIV clinic | *Probe about age, knowledge, self-management, knowledge about illness, knowledge about medicines* |
| 2 | What do you think can be done to ease the process of changing from health care providers for children to health care providers for adults in the HIV clinic for adolescents living with HIV? | Probes:  *How should be transition be handled*  *How should AYLHIV be empowered*  *What skills should be focused on to empower AYLHIV for transition* |
| 3 | What challenges are AYLHIV living with HIV likely to face as they change from health care providers for children to health care providers for adults in the HIV clinic? | Probes:  *Probe on each challenge raised and how they think these challenges can be solved* |
| 4 | As a parent/guardian what are your concerns about the change for AYLHIV living with HIV from health care providers for children to health care providers for adults in the HIV clinic? | *Probes*  *Probe on each concern raised and their suggestions on what can be done to address these concerns* |
| 5 | How can AYLHIV be empowered to gain the confidence they need to navigate the adult HIV clinic when they change from the children to the adult HIV clinic for continuity of care? | *Probe on*  *The social skills*  *The communication skills*  *Self-beliefs*  *Acceptance of change*  *Determination* |
| 6 | What challenges do AYLHIV face while interacting with adults patients living with HIV when they change care from the children’s to the adult HIV clinic | *Probe on each of the challenges raised*  *For those who have transitioned probe for specific challenges according to their personal experiences* |
| 7 | What challenges do AYLHIV face while interacting with health care providers in the adult HIV clinic when they change care from the children’s to the adult HIV clinic | *Probe on each of the challenges raised*  *For those who have transitioned probe for specific challenges according to their personal experiences* |
| 8 | What information do AYLHIV need concerning their illness before they change from health care providers for children to health care providers for adults in the HIV clinic? | *Probes:*  *Information on HIV, HIV medicines, benefits of medicines, side effects of medicines, adherence and retention in care* |
| 9 | What should AYLHIV living with HIV know about accessing HIV care before they change from health care providers for children to health care providers for adults in the HIV clinic? | Probes:  *Probe for why it is important for ALHIV to understand how to access HIV care services before they change care, where can they learn this information and at what age should they learn this information* |
| 10 | What are some of the things AYLHIV with HIV need to know about self-care before they change from health care providers for children to health care providers for adults in the HIV clinic? | Probes:  Probe on physical care, emotional wellbeing, social care |
| 11 | How should family members empower AYLHIV so that they can successfully change care from the children’s to the adult’s HIV clinic | *Probes:*  *Skills that AYLHIV can gain at home and community to enable them transition successfully* |
| 12 | How can health care providers empower AYLHIV to successfully change HIV care from the children to the adult HIV clinic? | *Probes*  *Skills AYLIHIV can get from health care providers to enable them transition successfully* |
| 13 | What capabilities do adolescents living with HIV need for a successful change from doctors for children to doctors for adults in the HIV clinic? | *Probes:*  *Explore from parents and guardians what ALHIV need to know before they change doctors and clinics, what they need to know about communicating with their health care providers, how they can learn these skills before the change process begins, at what age should they begin to get familiar with such skills* |
| 14 | What kind of preparation and training should AYLHIV receive before they change from health care providers for children to health care providers for adults in the HIV clinic? | *Probe on : What these trainings should comprise of, about what, at what age should they start, who should participate, who should facilitate these trainings, what would be the role of doctors and parents/guardians, how often should they happen* |
| 15 | What are some of the things that can be done to motivate AYLHIV to change from health care providers for children to health care providers for adults successfully | *For each factor mentioned probe why and who should be responsible to ensure that it happens* |
| 16 | What capabilities do AYLHIV need to cope with HIV as they change from the children’s HIV clinic to the adult HIV clinic? | Probes  *Probe on how they can seek support from health care providers and peers, how to express their feelings freely* |
| 17 | What capabilities do AYLHIV need to cope with the change of health care providers for children to health care providers for adults? | Probes  *Probe on how they can seek support from health care providers and peers, how to express their feelings freely* |
| 18 | How do you think AYLHIV can handle challenges that they may face during the process of changing from the children to the adult HIV clinic to continue HIV care? | *Probes*  *Communication*  *Knowledge about HIV care process*  *Information about navigating HV care*  *Social support systems*  *Self-disclosure* |
| 19 | What capabilities do AYLHIV need to ensure that the challenges they face when they change from the HIV clinic for children to the HIV clinic for adults will not affect their ability to continue with HIV care? | Probe on  Ability to focus on solutions  Determination to solve challenges  Confidence in self |
