# Supplementary material for: A qualitative analysis of self-management needs of adolescents and young adults living with perinatally acquired HIV in rural, southwestern Uganda
Source: PLOS Glob Public Health. 2024 Mar 18;4(3):e0003037. doi: 10.1371/journal.pgph.0003037 (PMC10947701; doi:10.1371/journal.pgph.0003037)
Supplement: S2 Text — (DOCX) [file pgph.0003037.s002.docx]

S2_Text

**Themes and Quotes related to the manuscript**

| **Themes** | **Quotes** |
| --- | --- |
| Communication between stakeholders | “You know you cannot do this without the support from the parents even if the child is at school the parents are involved and we see that something should be done like this and we really exchange our views and agree on what to do.”  “The parents and care givers should do the same like health care providers; sensitize their children about the safety of the medicine they are yet begin and this causes adolescents to adhere well to the medicine without fear. You know when you do something while panicking it sometimes causes problems, but if a parent comforts the child about the safety the transition, then the child will comfortably transition without any fear.”  “Actually it would be better before the transition the parents should be called together with the adolescents for sensitization so that whatever is being taught in case the adolescents forget the parent could remind them; some adolescents might be dull but if they are trained with their parents it is better and I think the parents should be present at the time of transition.”  “The doctors in the adult’s clinic should first see how we have been treated previously and may be tell us to adhere to the new system and be ready to face the challenges that may be there. The adults should also receive us and orient us and not just doing things as usual as if we have been there.”  “The family members can come to the clinic for guidance; they could work together with the health care providers so that to get some guidance on how to change their adolescent.” |
| Resilience | “We need to consider resilience as a life skill and self-esteem mainly because you find the reasons they give not to be sitting in the adult clinic is like these people are judging us right from the fellow clients; they are judging us they look at you and they are like eh even this young person is living with HIV? So that scares them but if they are resilient enough to all that stigma they can handle. And also self-esteem because if you have that self-esteem really the stigma, the external stigma will not affect you a lot.”  “It is all about perseverance because if they have reached the age for transition definitely they will find adults who have also persevered who may guide them and tell them that that is how things are done that side.”  “They need to persist because they have nothing else to do; they persist and get what they need and if talked to in a rude way, they could talk to the parents who also give them more encouragements.” |
| HIV status Disclosure | “Adolescents may not disclose their health challenges with the doctors at the adult clinic because of lack of trust and they end up keeping their burning and hurting problems to themselves.”  “Lack of privacy; those adult patients talk a lot; they will spread rumors about the children. When they see them at the clinic they will tell everyone in their communities how someone’s son/daughter has HIV. These children will feel uncomfortable interacting with those people because they don’t trust them at all.”  “Let me tell you, there is a situation where you find you cannot take medicine because of the people you are staying with and you don’t want them to know that you are sick and that makes you take medicine without water.”  “Sharing experiences with people you are not used to; it becomes more difficult because you don’t know the person you are talking to, but in the children’s clinic you find that you have been with someone for about 15 years, 10 years or 5 years and now you are dealing with these people in the adult clinic for the first time and when dealing with the person for the first time you don’t know anything about him/her meaning it will be difficult for you to disclose any information like you do in the children’s clinic.”  “As positive children they have to get a friend especially while at school and disclose to him or her so that in case they forget to take medicine, a friend can remind them. However, keeping everything confidential means that incase of anything there will be no one to help.”  “When they share their experiences and the time they have lived with HIV, they will learn that some have lived for more years than others, so they will have hope to live longer, even others that have never tested for HIV can pick courage to test and if they turn out positive they will join the services.”  “They need to get friends that are also positive because it is not good to disclose to everyone and let the whole village know that you are positive but if they are of the same status, they can share and help each other in case of any challenges.” |
| HIV stigma | “I think there will be still stigma like I have said they will be joining new people who are older than them like the ages of their grandparents, so to fit there it might be a challenge because they are used to their age mates and you know when you are with people of the same age you are more comfortable than when you are with different age groups. I think that is the only challenge I see.”  “Also there is always sort of stigma because when they reach in the adult clinic the way they have been interacting is not the same and sometimes they think that I might find here someone who knows me, my mother, my friend or a mother of my friend and she will tell her that I have seen your friend in the HIV clinic getting HIV medicines.”  “It is also there because sometimes you find a family of four children and only one child has HIV and there are also some families which are free whereby you find everyone knows that this particular child is HIV positive and some don’t know. So he/she feels stigmatized to find he/she is the only person sick in the whole family taking treatment and every time going to the clinic and that is from the family. At the clinic he/she might reach here and finds a school mate and when he/she finds a school mate sometimes he/she might end up going back because he/she definitely knows that this is an HIV clinic and that school mates will see and go telling others.”  “They are going to get stigma and they might not establish relationships like they did in the adolescent’s clinic where they sit together and talk about school, compare schools and laugh about it but in the adult clinic there is a lot to compare. So there will be no socializing, they will face stigma and they are going to be bombarded with a lot of new information because when we are giving health education they don’t say below 25 sit here, above 50 sit here; they give information to everyone as it is.”  “Also one of the reasons to why an adolescent may fail to transition to the adult clinic could be stigma because you see the adult HIV clinic is in the open space so there is no privacy whereas the children clinic is inside their triage is enclosed so there is privacy. That is why some of the adolescents fear that for us we are children if we go to the adult side we shall start meeting our relatives, neighbors. So stigma can be also a challenge to adolescents and youth to transition to the adult clinic.”  “They are going to be judged by the fellow adult clients actually because some of these people they seem not to know that we have mother to child transmission they only think about the horizontal transmission.”  “The bad and embarrassing comments from adult HIV patients; like I said they talk a lot, they are judgmental. They do not know how one contracted the illness but you will hear them throwing bad comments at you like surely at this tender age how did this girl contract HIV. You feel so discouraged and end up withdrawing from care.”  “And because of change of environment they are likely to also get stigma because of the people they will meet there. You find most of them know you and will disclose your status to others without your consent. Even it becomes a challenge interacting with adult patients because they talk too much; they are good at judging others. So they will judge them and that will also contribute on the stigma.” |
| Interpersonal skills | “They need life skills; I think life skills are very important skills to live with themselves, skills to live with other people and if they are able to communicate they will be able to maneuver through many things; how to live with yourself, how to live with others. Some of them you keep them to yourself but at times you have to share.”  “The children need to know that they have to be polite in speech because I know that when you politely ask someone for help they will respond to you. They should stop fearing any one because they are also human.”  “These children need to be taught how to handle themselves, how to express themselves freely, how to handle the difficult situation they might meet at the adult clinic. Also listen to their ideas, let them learn from each other by sharing their experiences. Let them know their rights and how they can fight for their rights like when not treated right at the adult clinic what they can do to get justice. Surely if that method is applied they will gain confidence and once they are changed to the adult clinic they will manage”  “The challenge they will face is this one; most of the adolescents have low-self-esteem and are very shy to interact with other people. So it becomes difficult for them to approach adult patients for advice. They fear them because of age difference. So that will make them feel lonely and they will hate themselves.”  “Their behavior, how they conduct themselves, how they take care of themselves should be considered also; so they should be well-behaved and mature enough to care for themselves, attend clinic appointments on their own without depending on their families all the time.”  “They should become friendly to those adults like I have been at children clinic; that will help me to learn more about the adult clinic and how to deal with some of the challenges I may come across.”  “They need life skills; I think life skills are very important skills to live with themselves, skills to live with other people and if they are able to communicate they will be able to maneuver through many things; how to live with yourself, how to live with others. Some of things you keep them to yourself but at times you have to share”.  “The children need to know that they have to be polite in speech because I know that when you politely ask someone for help they will respond to you. They should stop fearing any one because they are also human”.  “These children need to be taught how to handle themselves, how to express themselves freely, how to handle the difficult situation they might meet at the adult clinic. Also listen to their ideas, let them learn from each other by sharing their experiences. Let them know their rights and how they can fight for their rights like when not treated right at the adult clinic what they can do to get justice. Surely if that method is applied they will gain confidence and once they are changed to the adult clinic they will manage”.  “They should learn to interact with health care providers freely; build their confidence and approach them in case they need any service. They should also learn to be patient and remain calm; you see adult clinic has a lot of patients compared to the children clinic. And it seems doctors don’t keep time they always come late and they are a bit tough. So adolescents should learn to be patient and wait until they are worked on. They should not compare doctors and think that they will be treated the same way like at children clinic**”.** |
| Self-care | “Also the other skill they need I think would be self-care and positive living; it is so much important because this time they are going to be so much independent than previously when they were escorted by their caregivers, when the health worker was so much close to them and we were giving them a few months of refills to keep close to them, we were monitoring closely but if they have that skill of self-care and also they are responsible enough that means they can transition smoothly and everything will go on well.”  “Self-care is also a big component because it is about adherence, it is about personal hygiene, it’s about ability to interact a bit of personal relationship; how do you communicate with other people, how do you communicate with peers. So self-care is also very important.”  “He should be taking food and the medicine in time, avoiding much stress; thinking about why he is like that and as a parent you counsel and let him know that he is not the only one in that situation and it happened not by own making. I actually said all that to my first son and eventually he got used and that is how it is.”  “Self -care means a lot; so they should take their medicine well to have good health that is self-care and whatever you are doing will be successful because you have good health. They should accept their HIV status and express themselves freely that is self-care because once you accept, nothing can ever bother you; no stigma nothing can affect you once you accept who you are.”  “They should know that it is all about their lives and whatever they do should be for the good of their lives and that there is no one who is going to love them more that they do to themselves. They have to take care of themselves better than the way the doctors are doing it to them. They should love themselves and take good care of themselves by taking well their medicine, eating well, keep happy and think about their future so that whatever change is for their good of their lives. I think that is what they should know.” |
| Empowerment | “And another thing apart from age and emotional stability we need to make sure that they also have financial capacity. Yeah it should also be considered because currently someone might be making decisions for them; go to hospital here is transport. Now if you become an adult sometimes it might not be easy you might lose some of that and especially if you have not got a job or something to support you financially, we might lose you and at the end of the day you fail to come because your financial capacity is still very low and I think for me those are some of the things that should be looked at.”  “So financial stability we have to start working on it early by building professionalism within young people by giving them education which is very important because education opens many avenues and it doesn’t mean that what you study is what will make you financially stable NO but it will open your mind to other doors; yes I went to school thought I will be a teacher but when I reached there, I realized that I can study and the information I got, I got friends now I am investing in something else and that is what is making me financially stable.”  “Now the skilling of course we need to look at developmental components of skilling; they have to build skills of how to communicate, how to relate and how to do certain things so that they can negotiate a lot of things especially in their favor; they can learn to say “NO” or “YES” in their favor so that they are not harmed and other skills of survival, working and all that, but I also think that giving them skills to help them transition into adulthood, we need to give them skills that will help them especially when it comes to communication, they should be as assertive as possible so that they are not trampled on”  “If you have money you need to educate your child; you can take them to technical institutions if you have no money to pay for the university. You facilitate them to learn technical skills, which is not so difficult but even if it is difficult they can be supported because they are like any other children.”  “Because of age difference, they may face a challenge of disclosing their health challenges because their level of understanding is difference. So they will think that these adult patients will judge them so they will opt to keep everything to themselves. Also the conversation may not match because of age; those adults already have families, they have family challenges so they share with those on the same level for some advice not adolescents who don’t know anything about marriage.” |
| Self-advocacy skills | “I think the most important is for adolescents to be assertive it is really an important issue if you don’t know where to start from you will be able to walk to the health worker and ask where do I start from, if you have something to say you want to know your viral load, you want to be timid even though the clinician in front looks intimidated you will still ask, if you have a complaint and it hasn’t been addressed you will still ask, you will still communicate so to be assertive, confident I guess yeah.”  “They have to be very assertive; they have to ask questions like you have to encourage them to ask questions. You see if someone asks a question, if someone is very assertive you get to know what they are thinking.”  “Through being assertive; they communicate to their health care providers like you know this and this we are not comfortable with it and also to make sure that basically whatever happens in their life they report to the health worker because that is the most important thing something they can handle with the stress I mean the judgment from the adult client that is something they might not be able to change but if they report to their health worker maybe they can incorporate it into their health education and also being resilient.”  “Also these children should be left alone and exercise their rights; as parents we should not overprotect them. We should leave them to freely express themselves that is how they will gain confidence. Let them be because when you overprotect them they become insecure, they will never believe in themselves and they will never mature. So they need their space to exercise their rights and express their feelings freely.”  “They should be confident enough to consult the doctors where necessary. They should not fear them because those are their doctors that will attend to them forever. So they should make sure they become free and contact them for assistance. Also they should feel free to disclose because doctors are professionals they will not mistreat them.”  They need to believe in themselves; yeah let them believe in themselves in whatever they think is right they should do it. Also they need to be self-driven and they need to communicate too in case of any challenges, any difference let them speak out.  “Like I said they need to be empowered with the following; self-confidence, self-brief, acceptance of their HIV status, communication skills. If they can gain all these skills I believe they can handle any challenge either associated with their illness of health at large.” |
| Communication skills | “So all you need to do is to meet these children and teach them how to express themselves freely, how to handle the situation, where to report in case of anything and how to stand for their rights and speak out when not treated right. That is it. They should know that change is change; time will come when they will need to change to the adult clinic and they should accept once it happens.”  “I think communication is important like you talk to them, you engage them and with communication also you have to listen to them, you explain to them and if they have got issues listen because you may force someone and when s/he comes to the adult clinic they fail or decide to run away from care so I think communicating to them, talking to them would be very important.”  “Communication; the best way to handle challenges is to talk because those challenges are only going to be taken away by communication at different stages and different places; at school talk, at home talk, when you come here at the clinic talk. I have a challenge with the medicine when I take it I get nausea, when I take it I feel dizziness talk because some of the things may be could be social challenges, some could be with side effects of the medicines. So I think talking can actually solve the challenge.”  “There are those communication skills they need to learn, communication skills are so many you see for them they are used of talking anyhow, but when you crossover to the adult side, doctors take you to be an adult person you have to learn how to communicate with others because when you come and communicate to them badly that means they are going to blast you and when they blast you that means someone is going to get biased and will never come back to the facility. So they need some bit of communication skills; you know if you talk well to someone you get what you want.”  “Of course communication is key because information flows and then things are rectified in one way or the other. Then the other skill we talked about being able to start up small income generating activities if they are able and also the personal skills of being responsible enough and embrace the positive living. Even the social support systems that are available; the family, the peers, the friends.”  “Like I had said they need to have communication skills; they need to communicate well, they need to be able to explain themselves in terms of what they want, in terms of how they are feeling if they are facing any sickness they should be able to explain it to whoever they have encountered with that day.”  “Yes, communication and sharing with them could be one way of dealing with problems.” |
| Coping skills | “They should believe in themselves, they should accept as in accept that yes this is me I am HIV positive and this is how I was born. So they should accept that they are HIV and that will not change because if they don’t believe and if they don’t accept then that is very bad. Also they should learn to ask questions you see me I emphasize on communication skills and they should learn to be independent.”  “It is about believing in yourself; like I had said before, to have a belief that even though I am a patient but I am a person like any other and I am able. But this depends on their parents and the counseling they receive. The healthcare providers should add on the parents’ efforts and you, the healthcare provider? Some parents have no time to speak to their children. I for example look up to this one (the child), when I come I am the one who talks to her but for the mother, it is none of her business and it is not that she doesn’t want but that is her nature.” |
| Emotional Support | “When you counsel them and tell them that they have been living with HIV for a long time, they will be fine they will gain the confidence to remain in care so that they continue living. If they accept their status they will take their medicine without fail.”  “If an adolescent needs support the relatives should come in to support and where these adolescents need psycho-social support the relatives should also be in line. One thing is this is someone who knows this person better, what this adolescent is going through and everything. So I think giving them that psycho-social support showing that they are with them though they are growing is very important. Yeah though you are growing we are still behind you and we are still supporting you in all ways.”  “Most people at home do not know the HIV status of these children for example at home it is my mother, my wife and I that know the HIV status of our children, so if I am not around they can help give the child transport, food and also keep guiding the children and telling them about this disease and the best way to continue living with it.”  “As a parent I will make sure that I take my child through orientation at the adult’s clinic, go with her and show her that we start from her and end here so that after some time I can leave her to come alone.”  “Even the adults in the clinic if we see that the children have come and may be need our support we should help them because they are young. We do not have to talk about them because it is God who knows about them, you never know it could be the parents who spread HIV to the child so do not conclude that they got it through sexual intercourse.”  “We should encourage them that please, since you have been transitioned to the adult’s clinic please make sure you go and be there to stay. We should also remind them to take their medicine in time.”  “Now like us the parents to always sit down with our adolescents and discuss with them; you tell them encouraging words and treat them well. I think that could also be helpful because us the parents should be the first counselors to engage them. You need to first have a conversation with them, comfort them so that they are not heartbroken.” |
| Information support | “We need to give them information on what they are going to face in the new clinic; information about the health care providers that they are going to face, the different care point that they are going to face, and maybe our expectations of them; what we expect out of them as people who have transitioned to a new clinic.”  This should be the responsibility of the parents; give them information about their illness, how they were born HIV positive and how they have progressed with the illness. How they started taking medicine from birth up to now. Tell them the truth that is the life they will live in until death. As a parent encourage the child to accept the status of living with HIV and disclose to the trusted ones for them to live freely. Encourage them to take their medicine well to live a healthy life and stay away from things that can affect their health like alcohol, smoking, peer pressure and focus on their studies for those in school and work for those already working for a bright future.”  “They also need the information which they will basically get through the health care providers and if they are able to get it through the internet because most of them get information from internet and where they feel they are not understanding still it comes back to communication; they communicate to their health care workers for help.”  We need to give them information on the benefits that are in the adult clinic. By the way the benefits are also there quiet many. So once they appreciate those benefits then they should be able to appreciate and even have the urge; one should be having the urge to leave children’s clinic and be able to face the adult clinic. So giving information on the benefits is very important.”  “Parents should sit with their children and share the good things at adult clinic like the benefits they will get once they transition. Advise them to take their medicine well to have good health. They should not force them to change, they should instead persuade them to change and see the good things like becoming independent and make their own decisions, become what they want to be.” |
| Financial/instrumental support | “Financial support from their caregivers such that they can continue with care they should not neglect their responsibility thinking that since they have transitioned they should do everything by themselves. Their families should continue supporting them as before. Also health care providers should continue encouraging them to take their medications no matter the challenges they may encounter in the process of change.”  “They can support them financially and make sure they come to the clinic where necessary to ensure that retention is 100%; they can check on their medicines. We always encourage parents that if possible go to the tins and count medicines yourselves, they can check on their return dates. They empower them by giving them transport to come back to the clinic and maybe trying to talk to them also about their health. Counselling is for anyone who thinks and contributes positively to another person and of course that one… now to a parent it is a responsibility so they can talk to them in case of any challenges they can address them if not then they can talk to us or bring them to us and we talk to them.”  To continue support though we say that for this person to transition they should be independent but still they need the support from their family members in terms of checking on them sometimes providing to them transport because even as adults sometimes they can be broke you don’t have any money for transport. So they need to continue supporting them financially and psychologically to make sure that they make it there until they are really stable. |
| Health caregiver support | “If it was possible maybe the clinic or the hospital would have some provision such that in case we identify those who are needy and sometimes they miss clinic because of lack of food and transport there would a small portion of finance which would maybe help those needy ones.”  “The other thing in terms of care we also orient them to the health care provider’s team in the adult clinic; we try to show them those people whenever they come, we show them where to start from, the person responsible and so forth. So the skill I may say is to help them to depend on themselves and know how to go through all those channels and we always give them our phone contacts so that in case they are stuck they can call and ask any questions they would wish to know about.”  “Friendly and responsive services; you don’t have to be harsh to these people and at the same time you don’t need to be so smooth. You need to be friendly but responsive. Where you think someone needs to be scolded do so, where you think someone needs to be appreciated do so. You need to consider their emotions as well and also their opinions is very important. And also giving them time; it is hard but if you try it can really work out.”  “As doctors they have to sit with the children and tell them about the transition. I think the doctors know better what they should tell these children. The doctors should make sure that they bring these children closer and love them.”  “The doctors can guide us in the best ways to work on our projects, if we come up with our own projects they can tell us how to work on them better and for the parents they should give us time and social support. If they tell us that it is something good, we shall also be motivated but if they demoralize us, we shall also pull out.”  “Maybe if they could introduce adult health care providers to these adolescents they are trying to transition such that when they reach there, they know the people to handle them, they don’t see strangers. That can help them to disclose freely like they have been doing at children clinic.”  “Health care providers should create good relationship with the adolescents. They should ensure that adolescents are willing to change and the doctors at adult clinic should be friendly. They should treat them as children not adult they have been seeing. If these adolescents are treated well I believe they will not have issues with the change.”  This change is not easy but I think as health care providers should know that if an adolescent leaves the children’s clinic, of course there a lot that is not known to him or her, so they need to be patient with these adolescents though it is a little hard. I think the doctors should take things easy in case one makes a mistake, they shouldn’t make it so complicated for the patient. Now you see when adolescents develop fear they think of going back to the children’s clinic which might disorganize the health care providers. When someone finds hard time at the adult clinic they tend to misbehave, there is need to simplify things. |
| Peer support | “I think when they are transitioning they should consider taking them with their friends because if you take me alone I will be lonely and opt to go back to the children’s clinic to be with my friends because they encourage us so much; so moving them with their friends should be considered because friendship matters a lot.”  “I think when they are transitioning they should consider taking them with their friends because if you take me alone I will be lonely and opt to go back to the children’s clinic to be with my friends because they encourage us so much; so moving them with their friends should be considered because friendship matters a lot.”  “The other thing that I see concerning the time when they have transitioned us and we are new in the adult’s clinic it will not be easy for us to mix with the adults. They need to find a way of fixing us. You know when we are children alone we don’t fear each other they need to get us a day for only the youth who have been transitioned to come to the clinic.”  “I would think of is grouping; like grouping them and know that they are not transitioning them individually but as a group. At least it can help them not to feel unwanted like asking questions to themselves like why me? I maybe 19 years and short when I am looking at someone who is 16 but tall and I am like but the other person is also old why am I the only one being transitioned? Much as we have prepared you, you have all the information but because you are going alone, it can also affect you but when you were prepared as a group and you are going as a group I think it can ease the process.”  They should be encouraged to get friends from the village that are also HIV positive to always talk to incase of any challenges. If some of their friends have experienced the same challenges they will share with them how they were able to overcome them. |
| Knowledge about HIV | “They also need to know about retention; what are we expecting out of you, how you can achieve good retention and just in case you are unable to come to the clinic how you do go about it. You just don’t need to disappear we have to keep the communication flowing.”  “They have to know that they are living with HIV and have to care about the effects of the illness, know that they have to take the medicine all the time and that it has to be taken at the right time as prescribed by the doctors.”  “They should know that their life depends on that medicine. They should look at people who refused to take ARVs and see how they are and then compare with those that take medicine. They will see that those who take medicine are really okay, they do their work, study and complete and so they have to understand that this medicine keeps them healthy.”  “They should know that they are HIV positive and they will live with the illness for life because HIV has no cure. They should know that the medicine they take is to make them live long so if they want to live a long life they should take their medicine properly and follow the doctor’s instructions very carefully.”  “They shouldn’t forget taking the medicine; they should know that they are sick and need to have love for them-selves, adhere well to the medicine, and fit in the society. Not to have hate for self, avoid feeling isolated. You see we usually disorganize the adolescents and they are not like us adults, when adolescents find out that they are living with HIV they begin to hate themselves and generally they get disorganized.”  “They should know that HIV is transmittable but before you transmit it to another person you should disclose to them. For example, before I got married I told my partner that I was HIV positive.”  “They should know that they are sick and should protect their lives by taking the medicine and also protect themselves from spreading HIV to other people.” |
